# The Baby‐Friendly Community and Health Services Initiative Within the Joint Action PreventNCD: Rationale, Structure and Methodology

**DOI:** 10.1111/mcn.70229

**Published:** 2026-10-15

**Authors:** Francesca Zambri, Flavia Splendore, Vincenza Di Stefano, Anne Bergljot Bærug, Gry Hay, Ann‐Magrit Lona, Hanne Christine Mosand Bliksås, Gabriella Tambascia, Annachiara Di Nolfi, Tjaša Bonča, Marta García Sierra, Ieva Gudanavičienė, Nataliia Usenko, Venetia‐Maria Vraila, Angela Giusti

**Affiliations:** ^1^ National Center for Disease Prevention and Health Promotion National Institute of Health Rome Italy; ^2^ Department of Biomedicine and Prevention University of Rome Tor Vergata Rome Italy; ^3^ Norwegian Directorate of Health Oslo Norway; ^4^ National Institute of Public Health Ljubljana Slovenia; ^5^ Epidemiology and Public Health Area, FISABIO Foundation – Public Health Valencia Spain; ^6^ Public Health Department Ministry of Health of the Republic of Lithuania Vilnius Lithuania; ^7^ Public Health Center of the Ministry of Health of Ukraine Kyiv Ukraine; ^8^ Department of Social and Developmental Pediatrics Institute of Child Health Athens Greece

**Keywords:** baby‐friendly community, baby‐friendly initiative, breastfeeding, community health services, health equity, JA PreventNCD, non‐communicable diseases

## Abstract

The first 1000 days of life are a critical period for establishing lifelong health and development. Breastfeeding, as part of the Nurturing Care Framework, provides unique immunological, nutritional, and psychosocial benefits, while also contributing to the prevention of obesity, diabetes, and other non‐communicable diseases (NCDs). Despite its well‐documented advantages, breastfeeding rates remain suboptimal across Europe due to systemic barriers such as insufficient parental leave, aggressive marketing of breastmilk substitutes, limited specialised training in breastfeeding support within health services and inadequate community support. Within the European Joint Action Prevent NCD (GA No. 101128023), Task 6.5 ‘Baby‐Friendly Community and Health Services (BFC&HS)’ addresses this gap by implementing and scaling up the Norwegian best practice ‘Baby‐Friendly Community Health Services’ (BFCHS) in seven countries (Italy, Norway, Slovenia, Spain, Greece, Ukraine and Lithuania). The initiative applies to community and primary care settings, workplaces, educational institutions, and emergency response systems, with a focus on equity and vulnerable populations, through proactive outreach to underserved families, adaptation of services to local social and cultural contexts, coordinated care pathways between maternity and community services, and systematic monitoring of access and breastfeeding outcomes across key social determinants. The Task BFC&HS is structured into five subtasks: preparatory context analysis and stakeholder engagement; implementation of the BFCHS guidance through a staged Baby‐Friendly designation process; capacity building via training and e‐learning; creation of breastfeeding‐friendly environments; and promotion of the Nurturing Care Framework. Methodology emphasises a settings‐based, multisectoral approach rooted in the Ottawa Charter, combining evidence‐based standards, monitoring with WHO/UNICEF indicators, and transnational knowledge exchange. By integrating breastfeeding promotion within broader health strategies, the Task aims to embed supportive environments, strengthen local health systems, and reduce inequities and to contribute to the development and testing of a potentially scalable model for addressing NCD prevention and health equity through breastfeeding promotion across several European contexts.

## Summary

- Baby‐Friendly Community and Health Services approach protect, promote, and support breastfeeding while address health inequities beyond the child‐maternity healthcare. Extending the Baby‐Friendly Initiatives to community and primary health services is essential to ensure continuity of care after hospital discharge.
- The Baby‐Friendly Community and Health Services best practice provides an evidence‐based framework for translating WHO/UNICEF standards into community policy and practice.
- Task 6.5 of the Join Action PreventNCD demonstrates how multisectoral collaboration, standardised indicators, and capacity building can support the scalable implementation across diverse European contexts.
- Integrating breastfeeding into health systems, community environments, and policy frameworks can strengthen equity‐oriented public health strategies and sustainable non‐communicable diseases prevention.

## 1 Background

The first 1000 days of life, from conception to 2 years of age, represent a critical period for shaping lifelong health and development (WHO, UNCF & WBG 2018; Black et al. 2017). During this window, proper nutrition, including breastfeeding, lays the foundation for optimal physical growth, cognitive development, and immune system maturation (Pérez‐Escamilla et al. 2023). Breastfeeding is integral to this framework, providing unique immunological protection, reducing the risk of non‐communicable diseases (NCDs) later in life, and fostering strong maternal‐infant bonds (Rollins et al. 2016; Victora et al. 2016; Victora et al. 2021; Liu et al. 2025; Patnode et al. 2025). In addition to its direct health benefits, breastfeeding has ecological advantages by reducing the environmental and climate impact associated with Breast Milk Substitutes production and use (World Health Assembly Resolution WHA 2018; Horta and Victora 2013; GIFA 2023; Smith 2019; Dadhich et al. 2021; Pope et al. 2021). Promoting breastfeeding also aligns with broader public health goals to prevent social inequities, as suboptimal breastfeeding practices disproportionately affect vulnerable populations (Victora et al. 2016; Pérez‐Escamilla et al. 2023).

The importance of this period has been emphasised by the Nurturing Care Framework (WHO, UNCF & WBG 2018), which integrates responsive caregiving, adequate nutrition, and early learning, while also emphasising shared parental responsibilities and supportive family and social environments as key points for optimal childhood development and wellbeing. Cancer and other NCDs represent a significant portion of the disease burden in Europe, much of which is preventable (EC 2021, 2022; WHO 2024). The European Joint Action Prevent Non‐Communicable Diseases (JA PreventNCD; GA No. 101128023) is designed to address this challenge by supporting strategies and policies that aim to reduce the burden of cancer and NCDs, focusing on both personal and societal risk factors (JA PreventNCD n.d.). Breastfeeding, recognised as a cornerstone for preventing obesity, diabetes, and other NCDs, and identified as a cost‐effective public health ‘Best Buy’ intervention (WHO 2024, 2025; Binns et al. 2016; Collaborative Group on Hormonal Factors in Breast Cancer 2002; Su et al. 2021), continues to be practiced at lower‐than‐optimal levels in Europe, where rates are among the lowest globally (WHO & UNICEF 2019; UNICEF 2019). Despite robust evidence of its benefits, systemic barriers such as insufficient maternity leave (Baker et al. 2023), pervasive marketing of breastmilk substitutes (WHO 2022; World Health Assembly 2016; Rollins et al. 2023), and, in some cases, inadequate support by healthcare systems and communities hinder its widespread adoption (Khatib et al. 2023). Although individual support is crucial, breastfeeding should be viewed as a public health issue that necessitates investment at a societal level (Brown 2017; Pérez‐Escamilla et al. 2023).

Within this context, the Joint Action Task 6.5 ‘Baby‐Friendly Community and Health Services (BFC&HS)’ focuses on the implementation and scaling up of the Norwegian ‘Baby‐Friendly Community Health Services (BFCHS)’, evaluated as a best practice by the European Commission (Stepien et al. 2022; Baerug et al. 2016). The Norwegian BFCHS model was developed as an extension of the WHO/UNICEF Baby‐Friendly Hospital Initiative (BFHI) to ensure continuity of breastfeeding protection, promotion and support after hospital discharge through community and primary healthcare services. The model is grounded in the WHO/UNICEF Baby‐Friendly standards and adapts the principles of the Ten Steps to Successful Breastfeeding to preventive maternal and child health services at the community level. Its settings‐based and multisectoral approach is also consistent with broader breastfeeding implementation frameworks, which recognise that breastfeeding outcomes are shaped by interacting structural, community, health system, workplace and individual determinants requiring coordinated interventions across multiple levels (Rollins et al. 2023; Pérez‐Escamilla et al. 2023). While the original Baby‐Friendly Initiative (BFI) focused primarily on hospital settings, the Baby‐Friendly Community (BFC) initiative aims to address this gap by extending breastfeeding protection, promotion, and support beyond hospital discharge to community health services, primary health care, and the broader community. Task BFC&HS seeks to co‐create and implement this practice using a settings‐based approach that involves all relevant stakeholders, with an equity focus and particular attention to the needs of vulnerable groups. The Task leverages the Baby‐Friendly framework to address these systemic issues through a multi‐level, evidence‐based approach. It aligns with strategic public health goals outlined in the WHO/UNICEF Global Strategy for Infant and Young Child Feeding (WHO & UNICEF 2003, 2018; WCRF n.d.; World Health Organization 2014). It recognises breastfeeding as both a public health intervention and a social justice imperative. By integrating health promotion with social determinants of health, the Task BFC&HS embodies a holistic approach to maternal and child health.

Task 6.5 BFC&HS is part of Work Package (WP) 6 ‘Healthy Living Environment’, which aims to support all partners in designing, implementing, and evaluating best practices and pilot actions across different settings, such as education, workplace, health, social services, and digital media. This broader framework promotes a multisectoral approach, ensuring the integration of interventions and fostering sustainable, equitable local governance for health (Peiró‐Pérez et al. 2025).

A key feature of this Task is the implementation of the BFCHS best practice in seven European countries (Italy, Norway, Slovenia, Spain, Greece, Ukraine and Lithuania). This best practice, showing significant improvements in exclusive breastfeeding rates (Baerug et al. 2016). However, the transferability of the Norwegian BFCHS model across European countries cannot be understood as a simple replication process. Norway benefits from contextual conditions that may facilitate implementation, including relatively high breastfeeding rates, strong welfare and parental leave policies, well‐established community maternal and child health services, and sustained public investment in preventive healthcare. These structural conditions differ substantially across JA participating countries. Within the Task, the Norwegian model is therefore treated as a transferable framework rather than a fixed intervention package. Core components considered essential across settings include adherence to the WHO/UNICEF Baby‐Friendly standards, continuity of care between maternity and community services, workforce training, monitoring systems, and protection from inappropriate marketing of breastmilk substitutes. Other components, including implementation pathways, governance arrangements, organisational models and community‐level activities, are expected to be adapted according to national and local health system contexts, resources and population needs.

The specific objectives of the Task BFC&HS are: (1) to implement and adapt the ‘BFCHS best practice’; (2) to enhance the capacity of healthcare providers, policymakers, and community actors through targeted training and e‐learning initiatives; (3) to create supportive environments for breastfeeding within healthcare and community services, workplaces, educational institutions, and emergency response systems; (4) to develop and implement robust monitoring and evaluation frameworks to measure the impact of interventions on breastfeeding rates and equity outcomes; (5) to promote a Nurturing Care Framework that integrates breastfeeding with other components of early childhood development, such as responsive caregiving and adequate nutrition; and (6) to foster trans‐sectoral collaboration among stakeholders to ensure sustainability and scalability of interventions beyond the duration of the project.

## 2 Development of the Task BFC&HS Structure

The structure of the Task BFC&HS and its five subtasks was defined during the proposal development phase of the Joint Action PreventNCD, before the start of country‐level adaptation and implementation. The design was based on the objectives of the EU4Health‐funded Joint Action, the overall WP6 framework on healthy living environments, the WHO/UNICEF Baby‐Friendly standards, and the Norwegian BFCHS model, which had been identified as a transferable best practice. After the start of the project, participating countries contributed to the adaptation and operationalisation of the planned activities through context analysis, stakeholder mapping, technical meetings and exchange within Task BFC&HS. This adaptation and implementation process allowed countries to tailor the BFC&HS approach to their national and local health system contexts, while remaining aligned with the common objectives and structure defined in the Grant Agreement.

## 3 Methodology

Task BFC&HS adopts a contextual, multisectoral and equity‐oriented methodology grounded in stakeholder collaboration, adaptability and health systems strengthening. The initiative combines context analysis, capacity building, organisational change, policy advocacy and knowledge exchange to support the adaptation and implementation of BFC&HS across heterogeneous European settings. A central component of the methodology is the assessment of local barriers, resources and implementation opportunities to tailor interventions to different health systems, social and community contexts. Stakeholder engagement activities involve healthcare professionals, policymakers, mothers, fathers, parent support groups, civil society organisations, NGOs and actors from the community, emergency and asylum seeker systems. At the same time, the initiative recognises that the effectiveness and inclusiveness of community engagement processes may vary substantially across settings depending on governance structures, resource availability, representation of vulnerable groups, and the capacity to sustain meaningful participation over time.

The methodology also includes the development of practical implementation tools designed to facilitate the integration of Baby‐Friendly standards into routine practice. These include guidance for BFCHS implementation, e‐learning platforms for pre‐service and in‐service training, protocols for infant and young child feeding in emergencies, and guidance on breastfeeding, breastmilk management and complementary feeding in daycare settings. Capacity‐building activities are intended to strengthen professional competencies, continuity of care practices and implementation readiness across community and healthcare services.

Monitoring and evaluation activities are based on WHO/UNICEF indicators to support comparability and continuous improvement, while recognising differences in data availability, monitoring capacity and health information systems across countries. In parallel, policy advocacy and transnational knowledge exchange aim to strengthen alignment between national policies and international breastfeeding standards, including the WHO International Code of Marketing of Breast‐milk Substitutes and the EU Blueprint for Action.

Long‐term sustainability and transferability are addressed through the integration of breastfeeding protection, promotion and support into local health systems, policies and community structures (Cattaneo et al. 2010). This includes fostering local ownership through collaboration with governments, communities and healthcare providers, while embedding continuous professional development opportunities for health professionals.

Task BFC&HS is based on the assumption that breastfeeding practices are shaped not only by individual choices, but also by interacting health system, social, cultural, commercial and, policy determinants (Rollins et al. 2023; Pérez‐Escamilla et al. 2023). Accordingly, the initiative combines capacity building, organisational change, supportive environments and, policy actions to strengthen the conditions that enable breastfeeding at the population level.

## 4 Structure of Task 6.5 BFC&HS

Task 6.5 BFC&HS is structured into five subtasks, each with specific objectives and a detailed implementation strategy to ensure robust outcomes (Figure 1).

**Figure 1:**
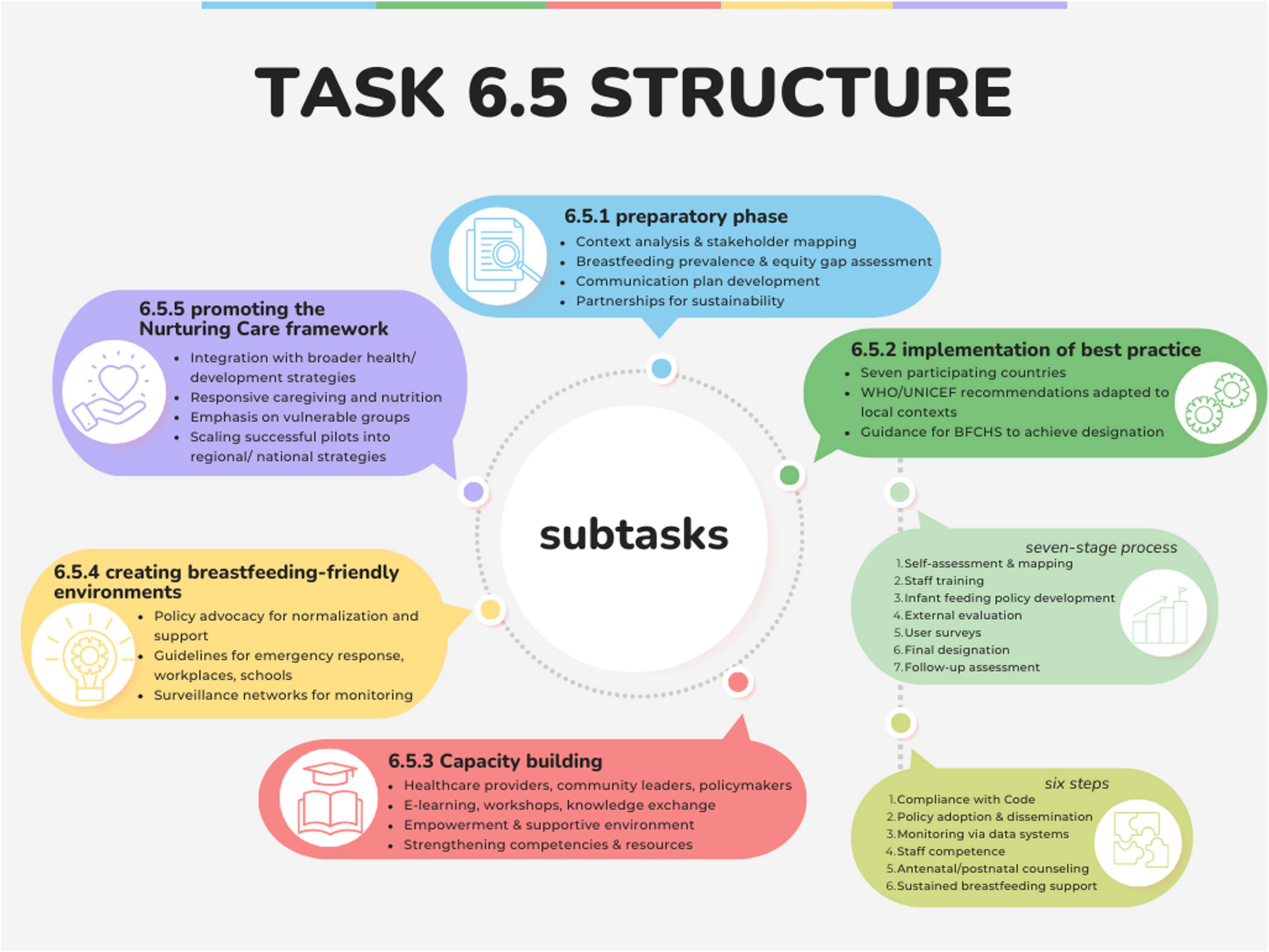
Task 6.5 BFC&HS structure: subtasks and activities.

Below, we outline the five subtasks. The *preparatory phase (Subtask 6.5.1)* establishes the foundation for adaptation and implementation by assessing local breastfeeding contexts, identifying equity and service gaps, and supporting stakeholder engagement and partnership building to facilitate adaptation, sustainability and local ownership. The *Implementation of best practice (Subtask 6.5.2)* focuses on the adaptation and implementation of the Norwegian BFCHS guidance (NDH 2025) across participating countries, using the WHO/UNICEF Baby‐Friendly standards as the core implementation framework. The guidance provides a structured pathway for designation that includes self‐assessment, workforce training, development of local infant feeding policies, external evaluation, monitoring, and follow‐up activities. Central components include compliance with the International Code of Marketing of Breastmilk Substitutes, strengthening professional competencies, continuity of care across maternity and community services, and sustained breastfeeding support for families. The implementation process is intended to support context‐sensitive adaptation of Baby‐Friendly standards across healthcare, community and workplace settings, while incorporating feedback and monitoring mechanisms to facilitate continuous improvement. *Capacity building (Subtask 6.5.3)* focuses on equipping healthcare providers, community leaders, and policymakers with the skills and knowledge needed to support breastfeeding and responsive parenting effectively within families and communities. Tailored e‐learning modules, workshops, and training sessions address identified gaps in breastfeeding knowledge, counselling skills and continuity of care practices among healthcare professionals and community actors. By strengthening professionals' confidence, communication skills and ability to provide consistent evidence‐based support, these initiatives are expected to improve the quality and continuity of breastfeeding support across services and settings. In addition, cross‐country knowledge exchange promotes shared learning and facilitates the adaptation of effective practices to different local contexts. *Creating breastfeeding‐friendly environments (Subtask 6.5.4)* involves advocating for policies, organisational practices and public communication strategies that normalise and support breastfeeding within everyday social environments, recognising that determinants operating at multiple levels influence breastfeeding practices. By increasing the visibility and social acceptability of breastfeeding in communities, workplaces, educational settings, digital environment and public spaces, these actions aim to reduce stigma, strengthen social support for families and facilitate breastfeeding as a socially supported and culturally accepted practice. Context‐specific guidelines and multisectoral partnerships are intended to promote structural changes that make breastfeeding support part of routine community life rather than an individual responsibility, also during emergencies, including through maternity protection, workplace breastfeeding support measures, and protection from inappropriate marketing of breast‐milk substitutes. Surveillance networks are established to monitor trends and inform policies. These environments are also intended to promote shared responsibility for infant and young child feeding and caregiving by actively engaging fathers, partners, families, employers and communities in breastfeeding support, thereby reducing the tendency to frame breastfeeding solely as an individual maternal responsibility. Finally, *promoting the Nurturing Care framework (Subtask 6.5.5)* integrates breastfeeding with broader health and development strategies. This framework (WHO, UNCF & WBG 2018) emphasises responsive caregiving, adequate nutrition, and early learning, particularly for vulnerable groups such as migrants and low‐income families. Successful pilot initiatives are scaled up and embedded into regional and national health strategies.

## 5 Impact and Benefits

The expected outcomes of Task 6.5 BFC&HS reflect its comprehensive and ambitious scope. By focusing on the adaptation and implementation of evidence‐based practices, the Task aims to create the conditions for improving breastfeeding rates across several contexts while addressing underlying health inequities.

This improvement is expected to stem from the integration of tailored interventions into healthcare systems, workplaces, and community settings. By providing supportive environments and enhancing awareness through education and policy changes, the Task BFC&HS seeks to normalise breastfeeding and empower mothers, fathers/partners and families to make informed choices.

Another expected contribution is to support efforts to reduce health disparities, in line with the overarching objective of JA PreventNCD to tackle social inequalities by addressing the root causes of NCD risk factors through a life‐course approach. By targeting vulnerable groups and adapting interventions to their specific needs, Task BFC&HS aims to ensure equitable access to breastfeeding support and resources. This focus also includes advocacy efforts for stronger regulatory measures at the EU level, both to promote breastfeeding in relevant policies and to protect it through regulating the marketing of breast‐milk substitutes, in line with the International Code of Marketing of Breast‐milk substitutes and subsequent resolutions. Addressing these systemic barriers is essential to achieving lasting health benefits for children and families.

Capacity building represents a cornerstone of Task's objectives. Strengthening the skills and knowledge of healthcare providers, with a particular focus on pre‐service and in‐service training, policymakers, and community actors will create a sustainable foundation for ongoing breastfeeding protection, promotion, and support. These efforts are expected to foster a culture of collaboration and shared responsibility among stakeholders, ensuring the scalability and longevity of the interventions.

Additionally, the development of robust monitoring and evaluation frameworks is anticipated to provide valuable insights into the effectiveness of the implemented strategies, generating evidence to guide future policies and programs.

Finally, the Task aims to contribute to a cultural shift that recognises breastfeeding as a public health and social priority. Through advocacy, education, and community engagement, Task BFC&HS aims to reshape perceptions and promote breastfeeding as a fundamental aspect of health and development. The ambition is to initiate a cultural transformation that will have ripple effects, influencing policies, practices, and behaviours well beyond the duration of the project.

## 6 Sustainability, Transferability, and Scaling Up

The task is committed to ensuring the long‐term sustainability of its initiatives by embedding breastfeeding protection, promotion and support into both national and local systems and policy. The project will build on the local adaptation and implementation of a best practice to scale up these efforts through collaboration with national and regional authorities. An important consideration for the transferability and scale‐up of the BFCHS model concerns the substantial heterogeneity across participating countries. Although all countries share common public health objectives related to breastfeeding protection, promotion and support, they differ considerably in terms of health system organisation, workforce capacity, governance structures, socioeconomic conditions and broader political contexts. Some participating countries benefit from relatively well‐established community maternal and child health services, while others face important structural challenges, including fragmented or overstretched primary care systems, regional inequalities, workforce shortages, and, in the case of Ukraine, the ongoing impact of armed conflict on health system functioning. These contextual differences are likely to influence not only implementation capacity but also the sustainability, scalability and equity of interventions. For this reason, Task BFC&HS adopts a flexible and context‐sensitive implementation approach, aimed at adapting shared Baby‐Friendly standards to different national and local realities. A further consideration supporting both sustainability and transferability is that the BFC initiative does not represent a novel undertaking in the participating countries. All partners have existing experience with the BFHI, and several have already extended this model to community settings through BFC programmes. This shared institutional foundation represents a considerable asset. The role of the Joint Action is therefore not to replace existing frameworks, but to build on them: drawing on the Norwegian BFCHS model, as a reference example of evidence‐based implementation, the JA offers an opportunity to define common minimum standards applicable across diverse national contexts. Equally important is the development of a structured mutual learning process among participating countries, through which different national experiences, implementation strategies and contextual adaptations can be compared and integrated. The ultimate aim is to foster a shared, evidence‐based vision of the BFCHS approach, one that is robust enough to guide policy convergence, while remaining sufficiently flexible to accommodate the realities of each health system. By establishing a shared monitoring and evaluation framework using WHO/UNICEF indicators, Task BFC&HS will provide a basis for expanding successful interventions across Europe and beyond, contributing to the global implementation of the WHO/UNICEF Global Strategy on Infant and Young Child Feeding. Monitoring and evaluation also represent important implementation challenges for BFCHS. Although Task BFC&HS promotes the use of standardised WHO/UNICEF indicators to support comparability across countries, the availability, quality and integration of breastfeeding data vary substantially between national and local health information systems. In many community settings, breastfeeding data collection may be fragmented, inconsistently recorded, or not routinely integrated into digital health systems. Furthermore, differences in indicator definitions, timing of data collection and reporting practices may limit cross‐country comparability. The implementation of monitoring activities may also place an additional burden on already understaffed maternal and child health services, particularly in settings with limited workforce capacity or constrained resources. These challenges highlight the need to balance robust monitoring systems with feasibility and sustainability considerations, while strengthening local data infrastructures and reducing unnecessary reporting burdens on healthcare professionals. This adaptive approach is particularly important when transferring best practices developed in high‐resource welfare contexts to countries with different health system capacities and social policy environments.

## 7 Integration with Other JA Work Packages

Task BFC&HS is closely integrated with the broader framework of the JA PreventNCD. Collaboration with other WPs and Tasks ensures coherence, sustainability, and scalability of interventions. In particular, Task BFC&HS aligns with WP4 (Sustainability), by ensuring that the results and outcomes are not only implemented at the local level but are also transferred and sustainably integrated into regional, national, and European policies. Furthermore, the synergy with WP4 contributes by identifying windows of opportunity within EU governance mechanisms, fostering synergies with other Joint Actions, and raising awareness among decision‐makers about the importance of mainstreaming integrated, sustainable approaches to health promotion in general and breastfeeding protection in particular. Collaboration with WP5 (Regulation and taxation) strengthens Task BFC&HS by addressing the broader regulatory and fiscal environment in which breastfeeding and child nutrition practices are framed, including regulatory measures consistent with the WHO International Code of Marketing of Breast‐milk Substitutes and subsequent resolutions (World Health Organization 1981). These efforts create a more coherent and enabling policy environment that reduces harmful marketing, promotes healthier food environments, and ensures consistency with breastfeeding protection and promotion strategies. This dimension is particularly relevant given the growing recognition of commercial determinants of health as major influences on infant and young child feeding practices. Evidence from the 2023 Lancet Breastfeeding Series highlights how the marketing strategies of the commercial milk formula industry can shape parental perceptions, social norms, health professional practices and policy environments, potentially undermining breastfeeding protection and support efforts (Rollins et al. 2023; Baker et al. 2023). In this context, adaptation and implementation of BFCHS requires not only strengthening healthcare and community support systems, but also addressing the broader commercial and regulatory environment in which infant feeding decisions are made. Integrating the WHO International Code of Marketing of Breast‐milk Substitutes within BFCHS implementation is therefore essential to ensure coherence between breastfeeding promotion policies and wider health system practices. Task BFC&HS activities are also integrated with WP7 (Social inequalities), through an explicit equity perspective in all breastfeeding promotion and support activities and environments. To operationalise this commitment, Task BFC&HS adopts the Health Equity Tool developed within the JA PreventNCD (Bucciardini et al. 2025). By embedding this tool into the planning and monitoring cycle of Task BFC&HS, the pilot action on BFC&HS is explicitly designed to reach and benefit population groups most exposed to inequalities in breastfeeding (initiation and continuation), including women in lower socioeconomic positions, migrants, and those living in areas with limited access to maternal and child health services. Moreover, WP8 (Monitoring) supports Task BFC&HS by strengthening the collection, integration, and analysis of data on breastfeeding, infant and young child feeding, and related health determinants. Building on the joint effort of participating countries and with the support of WHO Europe, a shared reflection is underway on existing national monitoring systems and on the feasibility of adopting WHO indicators (WHO & UNICEF 2021) in ways that would allow meaningful cross‐national comparability of breastfeeding data. Finally, synergies are also established with WP2 (Dissemination and communication) to guarantee consistent messaging and visibility of outcomes and activities.

Within WP6, Task BFC&HC is aligned with the common health promotion methodology developed through the Community Action for Health (CAH) framework (Peiró‐Pérez et al. 2025). This settings‐based approach, grounded in the Ottawa Charter, emphasises intersectoral collaboration, participatory governance, and equity, providing a structured 11‐step guide that spans preparatory, implementation, and evaluation phases, with an emphasis on conducting a needs assessment and health asset mapping in a participatory way, and co‐designing local interventions directly tied to the identified needs. By adopting this methodology, the Task ensures that breastfeeding promotion is integrated within broader community health strategies, closing the gap between research and practice and fostering sustainability. The CAH framework also facilitates learning across countries and settings, reinforcing Task as part of a ‘*European laboratory of health promotion experiences*’ (Peiró‐Pérez et al. 2025). This perspective highlights the complexity of translating Baby‐Friendly standards into sustainable community and health system interventions across heterogeneous European contexts. Strengthening the relevance and sustainability of Baby‐Friendly interventions therefore, requires continuous reflection, collaboration and long‐term institutional commitment.

## 8 Conclusions

Task 6.5 exemplifies a strategic approach to addressing public health challenges through the integration of evidence‐based interventions, stakeholder engagement, and trans‐sectoral collaboration. By sitting breastfeeding within the broader context of health equity and NCD prevention, Task 6.5 highlights the interconnectedness of biological, social, environmental and commercial determinants of health. The adaptation and implementation of the BFCHS best practice across seven European countries demonstrates its adaptability and potential for scalability. This Task provides a model for integrating protection, promotion, and support of breastfeeding into comprehensive strategies for achieving health equity and preventing NCDs, with implications that extend beyond Europe to global public health initiatives worldwide.

## Author Contributions

Francesca Zambri and Angela Giusti conceptualised the paper. Francesca Zambri wrote the first draft of the manuscript. Flavia Splendore, Vincenza Di Stefano, Angela Giusti and Anne Bergljot Bærug made major contributions to the revision of the manuscript. Gry Hay, Ann‐Magrit Lona, Hanne Christine Mosand Bliksås, Gabriella Tambascia, Annachiara Di Nolfi, Tjaša Bonča, Marta García Sierra, Ieva Gudanavičienė, Nataliia Usenko and Venetia‐Maria Vraila, contributed to critical revision of the manuscript. All authors read and approved the final version of the manuscript.

## Conflicts of Interest

The authors declare no conflicts of interest.

## Data Availability

Data sharing not applicable to this article as no datasets were generated or analysed during the current study.
